# Magnetic Particle-Based Automated Chemiluminescence Immunoassay for the Determination of Hydrocortisone Residues in Milk

**DOI:** 10.3390/foods14122105

**Published:** 2025-06-16

**Authors:** Yuan-Yuan Yang, Bao-Zhu Jia, Zhen-Lin Xu, Yi-Xian Liu, Lin Luo

**Affiliations:** 1 Guangdong Provincial Key Laboratory of Food Quality and Safety, College of Food Science, South China Agricultural University, Guangzhou 510641, China; yuanyuanyang2025@163.com (Y.-Y.Y.); jallent@163.com (Z.-L.X.); yixian_liu2022@163.com (Y.-X.L.); 2 College of Biology and Food Engineering, Guangdong University of Education, Guangzhou 510303, China; jbzjbz130@163.com

**Keywords:** chemiluminescence immunoassay, hydrocortisone, magnetic particles

## Abstract

Hydrocortisone is a typical glucocorticoid commonly used in livestock production; however, its overuse can result in hormone residues in milk. Long-term consumption of such milk may lead to a series of health issues. Therefore, the timely and rapid detection of hydrocortisone in milk is crucial for protecting human health. In this study, a magnetic particle-based direct chemiluminescence immunoassay (MP-DCLIA) incorporating a streptavidin–biotin signal amplification system was developed for the rapid and high-throughput detection of hydrocortisone in milk. Automated operations reduce human error and enhance the accuracy and repeatability of tests. The assay can be completed in 12 min with a linear detection range of 13.09–261.71 μg/L, a limit of detection (LOD) of 4.94 μg/L, a limit of quantification (LOQ) of 14.84 μg/L, and intra- and inter-batch variations of less than 5%. The method demonstrated stability and exhibited no cross-reactivity with structural analogues. Spiked recoveries of milk samples ranged from 85.85% to 100.30%, with results strongly correlating with those obtained from LC-MS/MS. The MP-DCLIA offers rapidity, high efficiency, stability, and precision, making it a promising tool for practical testing applications.

## 1. Introduction

Hydrocortisone, a representative glucocorticoid from the steroid hormone family, is characterized by its cyclopentane and polyhydrophenanthrene core structure [1]. This compound functions as an endogenous hormone produced by adrenal cells [2] and can be synthesized artificially for use in veterinary pharmaceuticals. Owing to its anti-inflammatory, antiviral, and anti-allergic properties, hydrocortisone plays a crucial role in regulating the synthesis and metabolism of lipids, carbohydrates, and proteins within the animal organism, thereby facilitating growth and reproduction. As a result, it is frequently employed in the context of animal husbandry [3,4]. However, the misuse or illicit application of such substances by unscrupulous vendors seeking to maximize profits can result in varying levels of hormone residues in animal milk. Chronic consumption of milk containing excessive hormone residues is associated with an increased risk of osteoporosis, obesity, hypertension, and other health complications [5,6,7,8], thereby posing a significant threat to public health. Numerous countries have established maximum permissible limits for hydrocortisone residues in food products. In China and the European Union, the use of hydrocortisone is restricted to external applications in edible animals. The United States, Canada, and Japan have established a maximum residue limit (MRL) for milk at no more than 10 μg/L. The current detection methods are characterized by cumbersome and time-consuming operations. Consequently, the development of a rapid and efficient method for detecting hydrocortisone in milk is of paramount importance.

Currently, the methodologies employed for the determination of hydrocortisone in food products include HPLC [9], HPLC-MS/MS [10], SERS [11], and ELISA [12]. Among these techniques, chromatographic analysis is noted for its high sensitivity and good reproducibility [13,14], making it the most widely utilized method. However, this technique imposes stringent requirements on the state of the sample to be analyzed, necessitating pre-treatment procedures such as SPE [15] and QuEChERS [16,17] prior to analysis. These procedures are highly specialized, which poses challenges for rapid, on-site testing of large sample volumes. SERS uses precious metals Ag and Au for signal enhancement, which is costly [18]. In contrast, ELISA, as an immunoassay method, offers advantages in terms of speed and cost-effectiveness; however, it is characterized by lower sensitivity and a narrower linear range compared to chemiluminescence immunoassay (CLIA). Moreover, natural enzymes are commonly used in ELISA, which have the disadvantages of poor stability, high cost, and harsh storage conditions [19]. CLIA effectively integrates high-sensitivity chemiluminescence technology with specific antigen-antibody binding reaction, enabling accurate detection of small molecules at low concentrations [20]. CLIA methods can be classified based on the chemiluminescent agent used. The CLIA method that employs acridine ester (AE) as a marker is referred to as Direct chemiluminescence immunoassay (DCLIA) [21]. After the immune reaction is complete, oxidants (e.g., hydrogen peroxide) and alkalis (e.g., sodium hydroxide) are added, and this chemiluminescent agent will be directly involved in the luminescence reaction, producing a light signal without the need for enzyme catalysis by the direct involvement of the chemiluminescent agent. This method has gained popularity as a rapid detection technique due to its high sensitivity, broad linear range, straightforward operational requirements, and no necessity for enzymes. It has become a significant technology in the domains of medical diagnostics, pesticide analysis, and veterinary drug detection [22]. Nevertheless, there is a paucity of literature regarding its application for the detection of hydrocortisone in food products.

In this study, the anti-hydrocortisone antibody labeled with acridinium ester (AE) as signal tracer was combined with a biotinylated antigen-streptavidin-magnetic particle conjugate to develop an automated direct chemiluminescence immunoassay. A magnetic particle-based direct chemiluminescence immunoassay (MP-DCLIA), utilizing acridinium ester (AE) as a marker, provides the advantages of low background luminescence and minimal interference from extraneous factors [23]. Additionally, the incorporation of a streptavidin–biotin signal amplification system, combined with the high-efficiency magnetic particle enrichment and separation system, enhances the assay’s sensitivity and precision. This approach also reduces reagent consumption compared to conventional microtiter plate-based CLIA, resulting in significant cost savings for detection [24,25]. Through the optimization of various reaction conditions, the established MP-DCLIA was successfully employed for the quantitative detection of hydrocortisone in milk. This advancement holds promise for the development of detection technologies for glucocorticoid drugs in food products of animal origin.

## 2. Materials and Methods

### 2.1. Chemicals and Apparatus

Zeba desalination column, magnetic particles (MP), and streptavidin were supplied by Thermo Fisher Scientific Co., Ltd. (Shanghai, China). Bovine serum albumin (BSA), dimethyl sulfoxide, and 2′,6′-Dimethylcarbonylphenyl-10-sulfopropylacridinium-9-carboxylate 4′-NHS ester (AE) were all provided by Sigma-Aldrich Co., Ltd. (St. Louis, MO, USA). Hydrocortisone sheep-derived monoclonal antibody (CORT-REAB-C1-001, 8.03 mg/mL), antigen, i.e., Hydrocortisone-BSA (CA00201B11, 3.38 mg/mL), excitation solution, and pre-excitation solution were supplied by GuangDong Fapon Biotech Co., Ltd. (Dongguan, China). Biotin, 1-ethyl-(3-dimethylaminopropyl) carbodiimide (EDC), n-hydroxysuccinimide (NHS), hydrocortisone, and prednisolone were supplied by Aladdin Biochemical Technology Co., Ltd. (Shanghai, China). Deoxycortisone, 17α-hydroxyprogesterone, dexamethasone, and prednisone were brought from Acmec Biochemical Technology Co., Ltd. (Shanghai, China). Corticosterone, progesterone, and cortisone were supplied by Yuanye Bio-Technology Co., Ltd. (Shanghai, China).

The rest of the chemical reagents were purchased from Guangzhou Chemical Reagent Factory. (Guangzhou, China). The following buffers were used: 20 mM of PB (pH 6.8); 50 mM of PB (pH 6.0); 25 mM of HEPES (pH 7.2); 0.5 M of Tris (pH 7.5); a blocking solution containing 25 mM of PB (pH 7.5), 1% BSA, and 0.5% Tween-20; PBS buffer containing 8 mM phosphate buffer (pH 7.2) and 25 mM sodium chloride.

Shine i2910 automatic chemiluminescence analyzer is provided by IncreCare Biotech Co., Ltd. (Shenzhen, China). BioTek Multi-function Enzyme Labeler was supplied by Agilent Technologies Inc. (Santa Clara, CA, USA). The laser particle size detector was supplied by Particle Sizing Systems. (Goleta, CA, USA). 6495D Triple quadrupole liquid-mass spectrometer from Agilent Technologies Ltd. (Santa Clara, CA, USA).

### 2.2. Preparation of Streptavidin-Coated MP

MP activation: 100 μL of MP stock solution was added to a 1.5 mL polystyrene centrifuge tube and placed on a magnetic frame to enrich the MP and remove the supernatant. Then, the MPs were washed twice with phosphate buffer, and the concentration of the MPs was adjusted to 10 mg/mL. Next, 100 μL of NHS (10 mg/mL) solution and 100 μL of EDC (10 mg/mL) solution were added and reacted at pH 6.0 and 25 °C for 30 min.

Streptavidin-coupled MP: The activated magnetic particles (MP) were magnetically separated, and the supernatant was removed. The particles were then washed with phosphate buffer (50 mM PB, pH = 6.0) and resuspended in PB. A specific amount of streptavidin was added to the suspension, and the mixture was rotated and oscillated at 25 °C for 2 h. Following this, the streptavidin-coupled MPs were magnetically separated and washed once with a blocking solution containing 25 mM phosphate buffer (pH 7.5), 1% BSA, and 0.5% Tween-20. The particles were then mixed with 1 mL of the blocking solution and incubated overnight in a rotary incubator at 25 °C with a rotation speed of 25 rpm. Finally, a preservation solution (0.5 M Tris buffer, pH 7.5) was added to the blocking solution to adjust the concentration of the coated MPs to 10 mg/mL. The prepared MPs were stored at 4 °C for future use.

### 2.3. Preparation of AE-Labeled Antibody

The Zeba desalting column was first washed with PBS buffer (8 mM phosphate buffer, pH 7.2, 25 mM sodium chloride). The antibody buffer system was then exchanged through desalting, and the antibody concentration was adjusted to 4 mg/mL. A specific amount of dimethyl sulfoxide was used to prepare a 10 mM AE solution, which was subsequently added to the antibody solution and allowed to react for 1 h at 25 °C. After the reaction, excess reagent was removed by desalting, resulting in the isolation of the AE-labeled antibody, which was stored at 4 °C.

### 2.4. Preparation of Biotinylated Antigen

The Zeba desalination column was washed with PBS buffer (8 mM phosphate buffer, pH 7.2, 25 mM sodium chloride). Subsequently, the antigen buffer system was replaced through desalination to adjust the antigen concentration to 4 mg/mL. A specific volume of 8 mM biotin solution, prepared with dimethyl sulfoxide, was added to the antigen solution and allowed to react for 1 h at 25 °C. After the reaction, the excess reagent was removed via desalination to obtain the biotinylated antigen, which was stored at 4 °C for future use.

### 2.5. Development of MP-DCLIA for Hydrocortisone Detection

**Figure 1:**
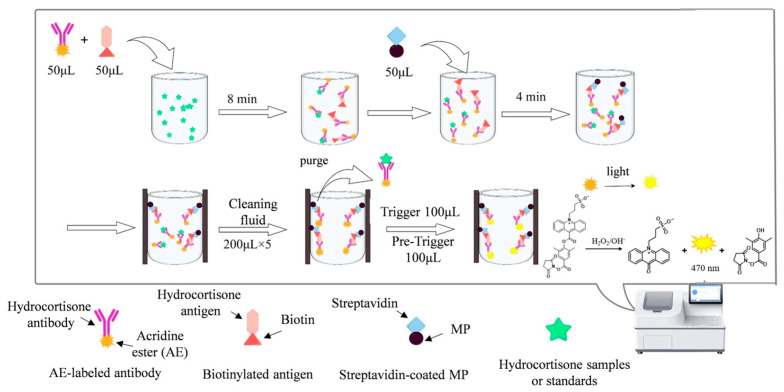
Schematic diagram of a magnetic particle-based direct chemiluminescence immunoassay (MP-DCLIA) for quantitative detection of hydrocortisone.

The procedure for quantitatively detecting hydrocortisone using MP-DCLIA is as follows: The three key reaction components—streptavidin-coated MP, AE-labeled antibody, and biotinylated antigen—are transferred to their respective reagent compartments. The reaction program is then configured on a fully automated chemiluminescence analyzer. Specific information on fully automated chemiluminescence instruments is in the Supplementary Materials (Figure S1). In the first step, 20 μL of the sample or a series of hydrocortisone standards, along with 50 μL of AE-labeled antibody (0.1 μg/mL, prepared in 20 mM PB at pH 6.8) and 50 μL of biotinylated antigen (0.05 μg/mL, prepared in 20 mM PB at pH 6.8), are added to the reaction cup for incubation, facilitating a competitive antigen-antibody interaction. The second step involves adding 50 μL of the streptavidin-coated MP (0.5 mg/mL, prepared in 25 mM HEPES at pH 7.2) for further incubation, resulting in the formation of a complex consisting of streptavidin-coated MP, biotinylated antigen, and AE-labeled antibody. In the third step, the complex is adsorbed, followed by washing (200 μL × 5) to remove unbound or non-specifically bound substances. Finally, 100 μL of pre-trigger solution (acidic solution containing 1% (*w*/*v*) hydrogen peroxide) and 100 μL of trigger solution (sodium hydroxide) are added. The corresponding relative light units (RLU) or specific concentration are then output by the automated chemiluminescence immunoassay analyzer (Figure 1).

Standard competition curves were generated for a series of hydrocortisone solutions in the optimal reaction mode using Origin Pro 2024b software. The linear range of the MP-DCLIA method was determined to be between IC_20_ and IC_80_.

### 2.6. MP-DCLIA Performance Evaluation

Limit of detection (LOD) and limit of quantification (LOQ): Ten consecutive independent replicate determinations were conducted using the zero standard. The mean (M) and standard deviation (SD) of the RLU values obtained from these ten determinations were calculated. Calculate the LOD and LOQ according to the following equations.

Equation (1): Limit of Detection (LOD) in MP-DCLIA

(1) LOD=M−3×SD

Equation (2): Limit of quantification (LOQ) in MP-DCLIA

(2) LOQ=M−10×SD

Specificity: Substances with a similar structure or function to hydrocortisone were selected for cross-reactivity. The specificity of the constructed CLIA method was evaluated by using cross-reactivity (CR) as an evaluation index, and the higher the cross-reactivity, the lower the specificity of the method.

Equation (3): Cross-reactivity (CR) in MP-DCLIA

(3) $$\mathrm{CR}=\frac{{\mathrm{IC}}_{50}\left(\mathrm{hydrocortisone}\right)}{{\mathrm{IC}}_{50}\left(\mathrm{cross}-\mathrm{reactant}\right)}\times 100\%$$

Stability: The stability of the MP-DCLIA was evaluated through accelerated thermal stability testing at 37 °C. Reagent vessels containing the three reaction components were placed in a 37 °C incubator for 0, 4, and 8 days. Afterward, the recovery samples with added milk were tested to compare the deviation in the measured values of the reagents following accelerated exposures of varying durations.

Accuracy: The precision of the MP-DCLIA established in this study was expressed as the coefficient of variation (CV) of the reagents on the measured values of the samples. Intra-batch precision: The sample was tested 10 times, and the mean value (M) and standard deviation (SD) of the 10 test results were calculated. Inter-batch precision: The sample was tested simultaneously 10 times with 3 batches of reagents. That is, 30 test results were obtained for the sample, and the mean (M) and standard deviation (SD) were calculated.

Equation (4): Coefficient of variation (CV) in MP-DCLIA

(4) $$\mathrm{CV}=\frac{\mathrm{SD}}{M}\times 100\%$$

### 2.7. Detection of Hydrocortisone

Sample Pretreatment: Ethanol is used to precipitate proteins, reduce matrix interference, and aid in the solubilization of extracted hydrocortisone [26,27]. In this process, milk is mixed with ethanol in a 1:2 volume ratio, vortexed for 3 min, and then centrifuged at 12,000 rpm for 5 min to promote precipitation. The supernatant is carefully aspirated, and this procedure is repeated twice. The supernatants from both extraction cycles are combined, evaporated to dryness, and reconstituted in a 1:1 ratio. Finally, the solution is filtered through a 0.22 μm membrane for testing.

For the recovery experiment, a known quantity of hydrocortisone was spiked into blank milk, and samples with low (10 μg/L), medium (100 μg/L), and high (200 μg/L) concentrations were subjected to the above pretreatment steps and analyzed by the MP-DCLIA. Milk samples were randomly collected from local supermarkets. The test solution was analyzed using both MP-DCLIA and LC-MS/MS to evaluate the accuracy and reliability of the MP-DCLIA, enabling a comparative analysis of results from both techniques. Specific details of the LC-MS/MS technique are provided in the Supplementary Materials (Table S1).

## 3. Results and Discussion

### 3.1. Characterization of Streptavidin-MP, AE-Labeled Antibody, and Biotinylated Antigen

**Figure 2:**
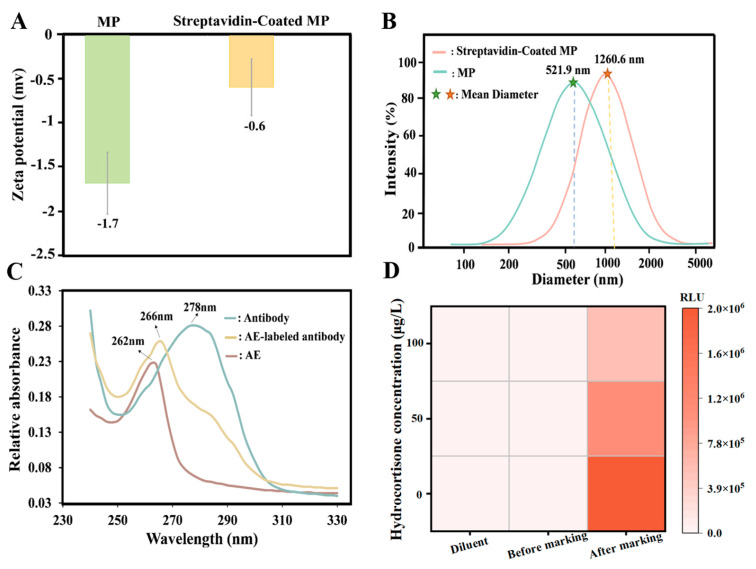
The characterization of streptavidin-MP, AE-labeled antibody, and biotinylated antigen. (**A**) Zeta Potential of MP and Streptavidin-Coated MP. (**B**) Particle Size of MP and Streptavidin-Coated MP. (**C**) UV-Vis Spectra of AE, Antibody, and AE-Labeled Antibody. (**D**) Changes in RLU before and after Antigen-Coupled Biotin.

Under specific conditions, the carboxyl group present on the MP can form a covalent bond with the amino group associated with streptavidin. As illustrated in Figure 2A, the zeta potential of the MP increased from −1.7 mV to −0.6 mV following the encapsulation of streptavidin. Additionally, the mean diameter of the particle size increased from 521.9 nm to 1260.6 nm (Figure 2B), indicating successful coupling of streptavidin with the MP.

As illustrated in Figure 2C, the characteristic absorption peak of the antibody is observed at 278 nm, while that of AE is at 262 nm, and the peak for AE-labeled antibody is at 266 nm. These findings suggest that the conjugation of antibody with AE results in a notable alteration in the spectral pattern, thereby indicating the successful synthesis of AE-labeled antibody.

As shown in Figure 2D, the RLU of the reaction system before coupling with biotin, as well as that of the system containing only the diluent (20 mM PB, pH 6.8), were notably low. In contrast, the reaction system following biotin coupling exhibited a marked increase in RLU, which then decreased as the hydrocortisone concentration increased. This indicates successful coupling of the antigen with biotin.

### 3.2. Optimization of the MP-DCLIA

To achieve optimal performance of the MP-DCLIA, we optimized the effects of antibody-antigen concentration ratios (4:3, 2:1, 4:1), competitive mode (simultaneous competition, sequential competition), and total incubation times (10 min, 12 min, 14 min, and 16 min) on the reaction system. In this case, the Antibody-antigen concentration ratios are 4:3 for 0.1 μg/mL:0.075 μg/mL, 2:1 for 0.1 μg/mL:0.05 μg/mL, and 4:1 for 0.1 μg/mL:0.025 μg/mL. The criteria for evaluating each influencing factor were the IC_50_ and the RLU_max_/IC_50_. A smaller IC_50_ value indicates better sensitivity of the method, while the magnitude of the RLU_max_/IC_50_ ratio determines the extent of the linear usable range of the curve [28]. Thus, among these conditions, those with lower IC_50_ values and higher RLU _max_/IC_50_ ratios would be selected.

**Figure 3:**
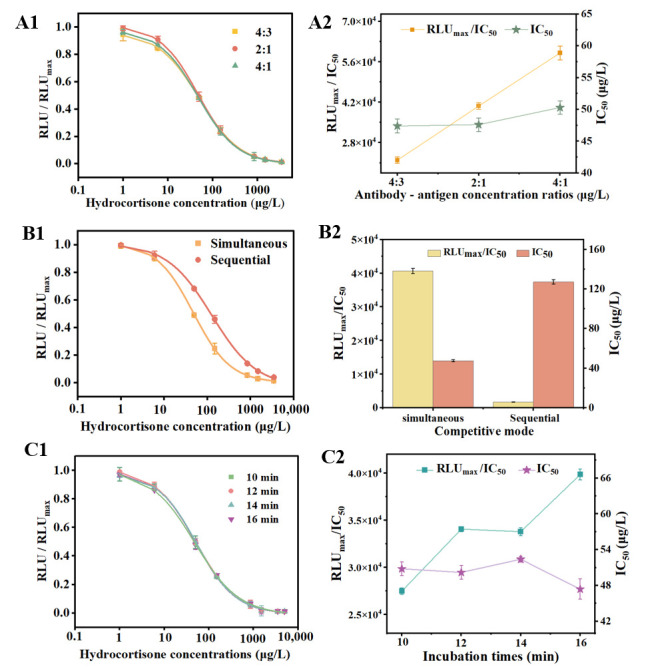
The effects of assay conditions on the performance of MP-DCLIA. (**A1**) Standard curves under different antibody-antigen concentration ratios (4:3, 2:1, and 4:1); (**A2**) IC_50_ values and RLU_max_/IC_50_ values under different antibody-antigen concentration ratios. (**B1**) Standard curves under different competition modes (simultaneous competition and sequential competition); (**B2**) IC_50_ values and RLU_max_/IC_50_ values under different competition modes. (**C1**) Standard curves under different total reaction times (10, 12, 14, and 16 min); (**C2**) IC_50_ values and RLU_max_/IC_50_ values under different total reaction times. Note: RLU stands for relative light units and refers to the RLU values for a range of hydrocortisone concentrations (excluding zero). RLU_max_ denotes the RLU value for a hydrocortisone standard at zero concentration.

Antibody-antigen concentration ratio: The precise recognition of the antigen by the antibody constitutes a critical step in the immunoassay process, and the concentration ratio of these two components significantly impacts the detection outcomes [29]. As illustrated in Figure 3A1,A2, when the concentration ratios of antibody to antigen were 2:1 and 4:1, the IC_50_ values for both ratios were lower and comparable. However, the RLU_max_/IC_50_ value for the 2:1 ratio was substantially higher than that for the 4:1 ratio. Therefore, it is concluded that the optimal reaction concentration ratio should be 2:1.

Competition mode: The inclusion of the biotinylated antigen in the initial stage of the reaction was intended to compete with the antigen present in the hydrocortisone standards or samples for binding to the AE-labeled antibody. This approach allowed for the classification of the competition mode into two categories: simultaneous and sequential competition. Figure 3B1,B2 shows that the RLU_max_/IC_50_ values for sequential competition were significantly lower, while the IC_50_ values were markedly higher than those for simultaneous competition. It is speculated that simultaneous competition mode allows for quicker reaction equilibrium, inhibiting biotinylated antigen binding to AE-labeled antibody at lower concentrations and providing more opportunities for complex formation. Consequently, it can be concluded that the optimal mode of competition is simultaneous competition.

Reaction duration: The duration of reaction time serves as a crucial metric for evaluating the detection efficiency of the method; typically, a shorter reaction time is desirable, contingent upon the preservation of detection performance. The findings presented in Figure 3C1,C2 demonstrate that the IC_50_ was 50.21 ± 1.16 μg/L for a reaction time of 12 min and 47.47 ± 1.70 μg/L for a reaction time of 16 min. The difference was not statistically significant; therefore, the method was primarily designed for high-throughput and rapid sample detection, leading to the selection of a 12-min reaction time. Consequently, the optimal total reaction time is established to be 12 min. The process was shorter compared to ELISA [30].

**Figure 4:**
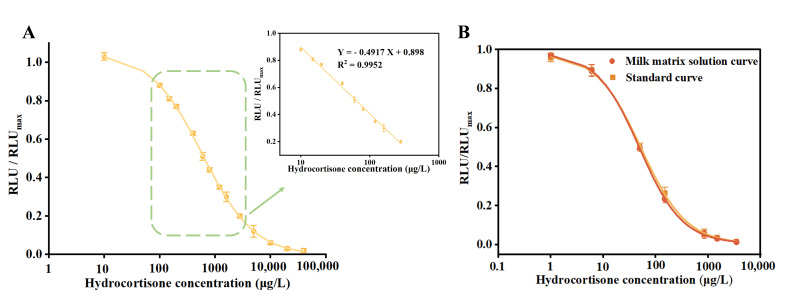
(**A**) The standard curve of MP-DCLIA for hydrocortisone (*n* = 3). (**B**) Comparison of the milk matrix solution curve and the standard curve.

A series of hydrocortisone standard solutions ranging from 0 to 4000 μg/L was prepared and tested under optimal reaction conditions to establish a standard competitive inhibition curve for the MP-DCLIA, as illustrated in Figure 4A. The IC_50_ for this method was determined to be 58.52 ± 1.81 μg/L, and the linear detection range (IC_20_~IC_80_) spanned from 13.09 to 261.71 μg/L. Compared to the Lateral Flow Assay Platform (LFAP), it has a broader detection range [31].

### 3.3. Performance of MP-DCLIA

The limit of detection (LOD) for the MP-DCLIA method was determined to be 4.94 μg/L, with a limit of quantification (LOQ) of 14.84 μg/L. In comparison to previous hydrocortisone assays, as illustrated in Table 1, the proposed MP-DCLIA exhibits lower LOD and comparable speed of testing. This efficiency is advantageous for achieving rapid and large-volume sample detection while providing superior detection performance.

**Figure 5:**
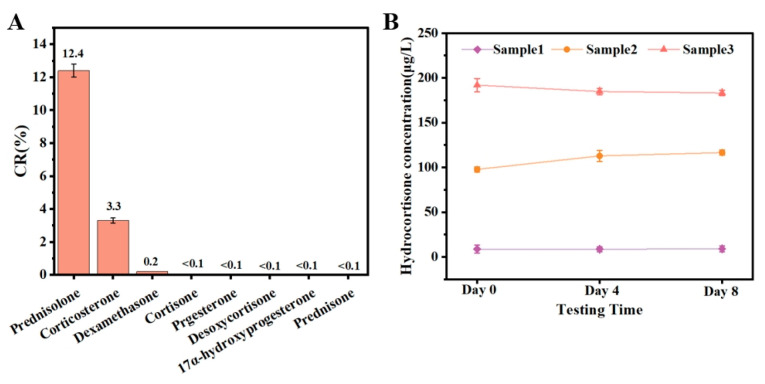
Performance of MP-DCLIA. (**A**) Cross-reactivity (CR) of analogues in MP-DCLIA. (**B**) The accelerated stability of MP-DCLIA.

The functional structural analogs of hydrocortisone were identified using the established MP-DCLIA method, and the cross-reactivity of each substance was assessed to evaluate the specificity of the method. As shown in Figure 5A, hydrocortisone exhibited a high cross-reactivity with prednisolone, a slight cross-reactivity with corticosterone and dexamethasone, and no significant cross-reactivity with the other analogs. This indicates that the established MP-DCLIA method demonstrates good specificity and is less susceptible to false positives.

The reagents were stored at 37 °C for 0, 4, and 8 days and were subsequently used to determine the spiked recoveries of the milk samples, as illustrated in Figure 5B. The results indicated that the measured values were consistent across all three reagents, despite the varying storage durations. This demonstrates that the MP-DCLIA method is stable and suitable for the routine determination of hydrocortisone in milk, yielding robust and reliable results.

Two different concentrations of hydrocortisone standards were evaluated to assess the precision of the MP-DCLIA. Figure S2 demonstrates that the intra-batch coefficients (10 tests) of variation (CV) for MP-DCLIA were 2.3%, while the inter-batch (30 tests) CV was 4.3%. Both values were below 5%, indicating that the established method exhibits good precision and that the correlation between results from different batches is strong.

### 3.4. Recovery Tests

Initially, the calibration curve prepared with the milk matrix solution was compared with the standard curve to evaluate the matrix effect. As shown in Figure 4B, these two curves almost overlapped, with their IC_50_ and linear detection ranges being close to each other, indicating that the matrix effect was eliminated. The spiked recoveries can be utilized as a criterion for assessing the accuracy of the method. Milk samples were spiked at low (10 μg/L), medium (100 μg/L), and high (200 μg/L) concentrations were subsequently analyzed using MP-DCLIA and LC-MS/MS. The results of these analyses are presented in Table 2. The recoveries ranged from 85.85% to 100.30%, and the coefficients of variation varied from 2.19% to 9.64%, all less than 10%. Furthermore, the results from the MP-DCLIA demonstrated a strong correlation with those obtained from LC-MS/MS, indicating that the accuracy of this method meets the requirements for determining hydrocortisone levels in milk samples.

### 3.5. Detection of Hydrocortisone in Commercial Milk

Commercially available milk was analyzed using LC-MS/MS in conjunction with MP-DCLIA, with an MRL of hydrocortisone set at 10 μg/L as the criterion for classifying samples as negative or positive. In this context, ‘-’ indicates a negative sample, while ‘+’ signifies a positive sample. The strong correlation between the results of MP-DCLIA and LC-MS/MS indicates that MP-DCLIA can be utilized as a reliable method for determining hydrocortisone levels (Table S2).

## 4. Conclusions

In conclusion, we developed a novel chemiluminescence immunoassay (MP-DCLIA) for the rapid and sensitive detection of hydrocortisone in milk samples. This method employs a streptavidin-biotin system, AE as a luminescent marker, and magnetic particles for target enrichment and impurity separation, enabling automated detection. Key parameters, such as reaction time and competition mode, were optimized to enhance MP-DCLIA performance. Under optimal conditions, the assay achieved a LOD of 4.94 μg/L, a LOQ of 14.84 μg/L, and a linear detection range of 13.09 to 261.71 μg/L, demonstrating high sensitivity for hydrocortisone. In our study, we have implemented and optimized the application of MP-DCLIA, which offers several advantages over existing methods. Unlike chromatography-based approaches, MP-DCLIA eliminates the need for complex sample preprocessing, thereby streamlining the workflow. Compared to traditional enzyme-based chemiluminescence immunoassays, MP-DCLIA requires less detection time and enables automated, high-throughput analysis. These improvements highlight the potential of MP-DCLIA as an efficient and practical alternative for detecting glucocorticoids in animal-derived food products.

## Figures and Tables

**Table 1:** Comparison of MP-DCLIA with other hydrocortisone assays.

Measurement Methods	Linear Range (μg/L)	LOD (μg/L)	Reaction Time (min)	Detect the Object	Reference
ELISA	0.10~2.00.	0.04	>90	cosmetic	[30]
Portable Chemiluminescence-Based Lateral Flow Assay Platform	0.78~12.5	0.342	13	Serum	[31]
Electrochemical biosensing platform	*	13.41	≥7	Wastewater	[32]
SERS sensor	36.25~3.63 × 10^5^	36.25	*	Saliva	[33]
LC-MS/MS	10~1000	10	≥11	Buffalo milk	[34]
Magnetic particles direct chemiluminescence immunoassay	13.08~261.71	5.45	12	Milk	This work

Note: An asterisk (*) indicates information that is not explicitly mentioned in the article.

**Table 2:** Milk sample addition and recovery (*n* = 3).

Addition (μg/L)	LC-MS/MS (μg/L)	MP-DCLIA (μg/L)	CV (%)	Recovery (%)
10	10.72 ± 0.91	8.75 ± 0.84	9.64	87.53
100	105.72 ± 0.74	100.30 ± 2.19	2.19	100.30
200	191.50 ± 1.93	171.71 ± 4.24	2.47	85.85

Note: The recovery ratio and CV are outcomes derived from the MP-DCLIA method.

## Data Availability

The original contributions presented in this study are included in the article/Supplementary Materials. Further inquiries can be directed to the corresponding author.

## Supplementary Materials

The following supporting information can be downloaded at: https://www.mdpi.com/article/10.3390/foods14122105/s1, Figure S1: Pictures of Fully Automatic Chemiluminescent Instruments; Figure S2: The inter-batch difference and intra-batch difference of reagents in MP-DCLIA; Table S1: Mass spectrometry conditions; Table S2: Results of hydrocortisone detection in commercially available milk.
