# Supplementary material for: Magnetic Particle-Based Automated Chemiluminescence Immunoassay for the Determination of Hydrocortisone Residues in Milk
Source: Foods. 2025 Jun 16;14(12):2105. doi: 10.3390/foods14122105 (PMC12191908; doi:10.3390/foods14122105)
Supplement: Supplementary file 1 [file foods-14-02105-s001.zip › foods-3615493-supplementary.pdf]

Supplementary material

Magnetic particle-based automated chemiluminescence immunoassay for the determination of hydrocortisone residues in milk

Yuan-Yuan Yang [1, #], Bao-Zhu Jia [2, #], Zhen-Lin Xu [1], Yi-xian Liu [1], Lin Luo [1, *]

1. Guangdong Provincial Key Laboratory of Food Quality and Safety, College of Food Science, South China Agricultural University, Guangzhou, 510641, China; lin.luo@scau.edu.cn (L.L.); nevertheless25@163.com (L.G.)

2. College of Biology and Food Engineering, Guangdong University of Education, Guangzhou 510303, China;

# Theses authors are equally contributed to this work

* Correspondence: lin.luo@scau.edu.cn (L.L.);

1、Specific Information on the Fully Automated Chemiluminescence Instrument

The Shine i2910, a fully automated chemiluminescence instrument utilized in this study, was manufactured by IncreCare Biotech Co., Ltd. (Shenzhen, China). This instrument comprises several key components: a sample handling module, a reagent handling module, a reaction cup transfer module, a sample reagent dispensing module, a reaction solution mixing module, a reaction module, a substrate module, a touch screen, and a software system.

The sample handling module is responsible for loading, transferring, and performing preliminary processing of samples. The reagent handling module is designed for the storage and management of reagents. The cup transfer module ensures the efficient transfer of cups between different modules. The sample reagent dispensing module allows for precise dispensing of samples and reagents. The reaction solution mixing module guarantees that samples and reagents are adequately mixed through various methods. The reaction module provides an optimal environment for completing the chemiluminescence reaction. The substrate module supplies the necessary substrate for the reaction. The touch screen serves as the operator interface, enabling users to control the instrument and view results. Finally, the software system manages the instrument's operations, oversees data management, and optimizes troubleshooting processes. Reason: Improved clarity, readability, and

technical accuracy while correcting grammatical and punctuation errors.

The instrument can accommodate 25 reagent containers and 60 reaction samples simultaneously, with a testing speed of approximately 200 tests per hour. Users simply need to configure the reaction program (including reaction duration, sample addition sequence, and sample addition volume), place the reaction reagents and samples in the designated positions, and click to initiate the reaction. The instrument will then output the corresponding RLU value.

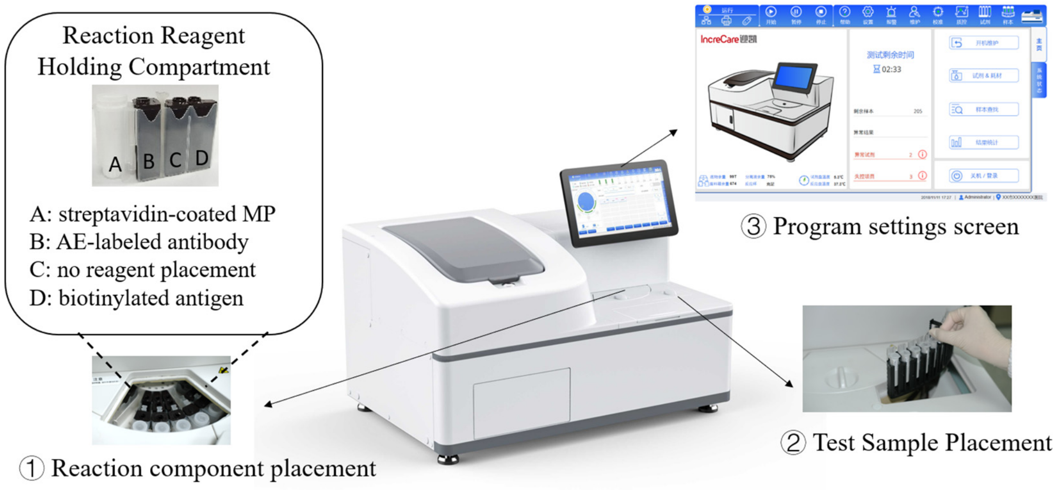

Figure S1. Pictures of Fully Automatic Chemiluminescent Instruments

2、The specific reaction condition parameters for LC-MS/MS were as follows:

(1) Composition of the mobile phase:

Mobile phase A: 0.5mmol/L NH_4F aqueous solution;

Mobile phase B: methanol.

(2) Chromatographic conditions:

Injection volume: 40 µL;

Flow rate: 0.2 mL/min;

Column temperature: 45°C;

Autosampler temperature: 10°C;

Column: Packing type C18, packing size 1.7µm, column size 2.1*100mm.

(3) Mass spectrometry conditions:

Table S1. Mass spectrometry conditions

Parametric	Setting
Ionisation mode	Electrospray ion source, positive and negative ions collected simultaneously
Detection method	Multi-response monitoring (MRM)
Capillary (kv)	2.5
Desolvation Temp (℃)	600
Desolvation (L/Hr)	1000
Cone (L/hr)	150
Nebuliser (Bar)	7.0
Collision Gas Flow (mL/min)	0.15

3、Accuracy results of MP-DCLIA

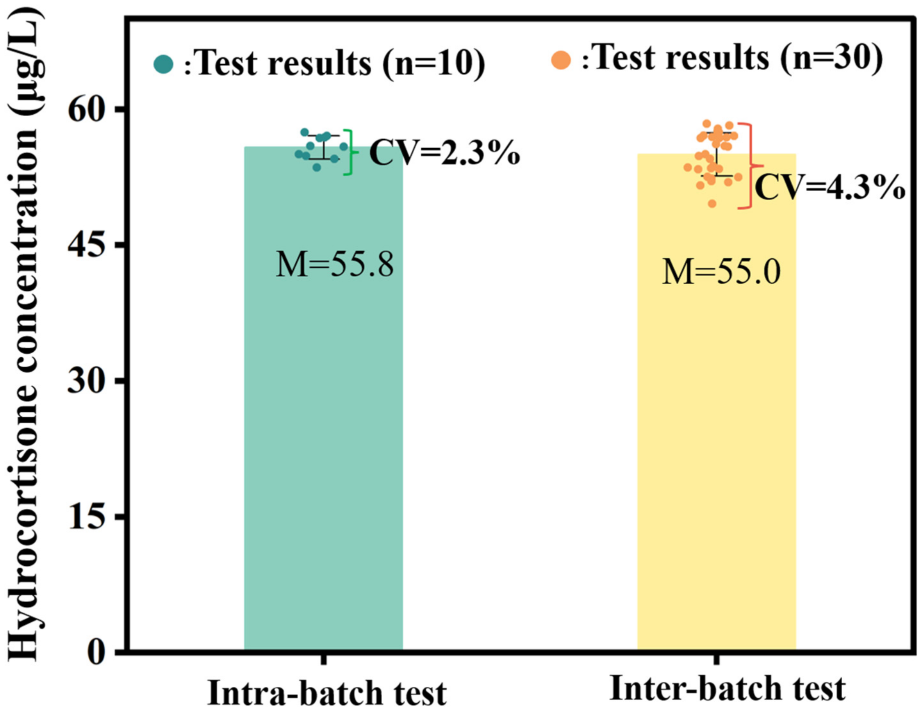

Figure S2. The inter-batch difference and intra-batch difference of reagents in MP-DCLIA.

4、Detection of hydrocortisone in commercially milk

Table S2. Results of hydrocortisone detection in commercially available milk.

Sample	MP-DCLIA	LC-MS/MS
1	-	-
2	-	-
3	-	-
4	-	-
5	-	-
6	-	-
7	-	-
8	-	-
9	-	-
10	-	-
11	-	-
12	-	-
13	-	-
14	-	-
15	-	-
16	-	-
